# Functional Characterization of *NIPBL* Physiological Splice Variants and Eight Splicing Mutations in Patients with Cornelia de Lange Syndrome

**DOI:** 10.3390/ijms150610350

**Published:** 2014-06-10

**Authors:** María E. Teresa-Rodrigo, Juliane Eckhold, Beatriz Puisac, Andreas Dalski, María C. Gil-Rodríguez, Diana Braunholz, Carolina Baquero, María Hernández-Marcos, Juan C. de Karam, Milagros Ciero, Fernando Santos-Simarro, Pablo Lapunzina, Jolanta Wierzba, César H. Casale, Feliciano J. Ramos, Gabriele Gillessen-Kaesbach, Frank J. Kaiser, Juan Pié

**Affiliations:** 1 Unit of Clinical Genetics and Functional Genomics, Departments of Pharmacology-Physiology and Pediatrics, School of Medicine, University of Zaragoza, E-50009 Zaragoza, Spain; E-Mails: eteresa@unizar.es (M.E.T.-R.); puisac@unizar.es (B.P.); mcgil@unizar.es (M.C.G.-R.); carobaque@gmail.com (C.B.); mhmarcos@unizar.es (M.H.-M.); ckaram78@yahoo.es (J.C.K.); milagrosciero2005@yahoo.es (M.C.); framos@unizar.es (F.J.R.); 2 Sektion für Funktionelle Genetik am Institut für Humangenetik, Universität zu Lübeck, D-23538 Lübeck, Germany; E-Mails: Juliane.Eckhold@uksh.de (J.E.); Diana.Braunholz@uksh.de (D.B.); 3 Institut für Humangenetik, Universität zu Lübeck, D-23538 Lübeck, Germany; E-Mails: andreas.dalski@uksh.de (A.D.); G.Gillessen@uksh.de (G.G.-K.); 4 Department of Pediatrics, Hospital Pablo Tobon Uribe, 05001000 Medellín, Colombia; 5 Institute of Medical and Molecular Genetics, Hospital Universitario La Paz, E-28046 Madrid, Spain; E-Mails: fernando.santos@salud.madrid.org (F.S.-S.); plapunzina.hulp@salud.madrid.org (P.L.); 6 Department of Pediatrics, Hematology, Oncology and Endocrinology and Department of General Nursery Medical University of Gdańsk, P80-211 Gdańsk, Poland; E-Mail: kwierz@gumed.edu.pl; 7 Department of Molecular Biology, Science School, National University of Rio Cuarto, 5800 Córdoba, Argentina; E-Mail: ccasale@exa.unrc.edu.ar; 8 Genetics Clinic, Service of Pediatrics, University Clinic Hospital “Lozano Blesa”, E-50009 Zaragoza, Spain

**Keywords:** CdLS, *NIPBL*, splicing mutations, physiological splicing

## Abstract

Cornelia de Lange syndrome (CdLS) is a congenital developmental disorder characterized by distinctive craniofacial features, growth retardation, cognitive impairment, limb defects, hirsutism, and multisystem involvement. Mutations in five genes encoding structural components (*SMC1A*, *SMC3*, *RAD21*) or functionally associated factors (*NIPBL*, *HDAC8*) of the cohesin complex have been found in patients with CdLS. In about 60% of the patients, mutations in *NIPBL* could be identified. Interestingly, 17% of them are predicted to change normal splicing, however, detailed molecular investigations are often missing. Here, we report the first systematic study of the physiological splicing of the *NIPBL* gene, that would reveal the identification of four new splicing isoforms ΔE10, ΔE12, ΔE33,34, and B’. Furthermore, we have investigated nine mutations affecting splice-sites in the *NIPBL* gene identified in twelve CdLS patients. All mutations have been examined on the DNA and RNA level, as well as by *in silico* analyses. Although patients with mutations affecting *NIPBL* splicing show a broad clinical variability, the more severe phenotypes seem to be associated with aberrant transcripts resulting in a shift of the reading frame.

## 1. Introduction

Cornelia de Lange syndrome (CdLS; OMIM 1227470, 300590, 610759, 614701, 300882) is a congenital developmental disorder characterized by distinctive craniofacial features, growth retardation, cognitive impairment, limb defects, hirsutism, and abnormalities of other systems with variable expressivity [1]. Mutations in five genes, encoding structural components of the cohesin complex (*SMC1A*, *SMC3*, and *RAD21*) and its regulators (*NIPBL* and *HDAC8*) have been found in patients with CdLS [2,3,4,5,6,7,8,9]. Cohesin was originally described for its function in regulating sister chromatid cohesion during mitosis and meiosis, but have also been demonstrated to play a critical role in DNA-damage repair and the regulation of gene expression [3].

Approximately 60% of patients with CdLS carry an identifiable mutation in *NIPBL* [2,4,5]. This gene is located on chromosome 5p13.2 and contains 47 exons. Thus far, only two splicing isoforms have been detected in embryonic tissues, although there may be more variants due to the large size of *NIPBL* [10,11]. In the main isoform (A), exons 2–47 codify for 2804 amino acids. The alternative isoform (B) does not include exon 47 and ends in an expanded variant of exon 46, codifying for 2697 amino acids. Both isoforms are conserved in vertebrates and are identical from amino acid 1 to 2683, while the *C*-terminal ends are different [11,12,13].

Currently, nearly 300 mutations in *NIPBL* gene have been reported, 49 of which affect splice-sites, representing 17% of total mutations [14,15]. However, systematic investigations on physiological splicing have not been evaluated in most of the cases. While truncating mutations are usually associated with a severe phenotype, and non-truncating mutations with a mild phenotype, clinical manifestations observed in patients with splicing mutations are highly variable in CdLS [4,16].

In this article, we have performed the first systematic analysis of the physiological splicing of *NIPBL*, which has yielded four new splicing variants. In addition, we have characterized the pathological splicing on twelve CdLS patients, in which we have identified seven new splicing mutations. This study was carried out using various molecular approaches on DNA, on RNA and *in silico* analyses.

## 2. Results

### 2.1. Clinical Findings

Clinical evaluation by at least two expert clinicians of all patients included in this study confirmed they meet the criteria for CdLS according to Kline *et al.* [1]. Molecular and clinical data relative to each patient are summarized in Table 1.

### 2.2. Aberrant Splicing Caused by NIPBL-Mutations

**Figure 1:**
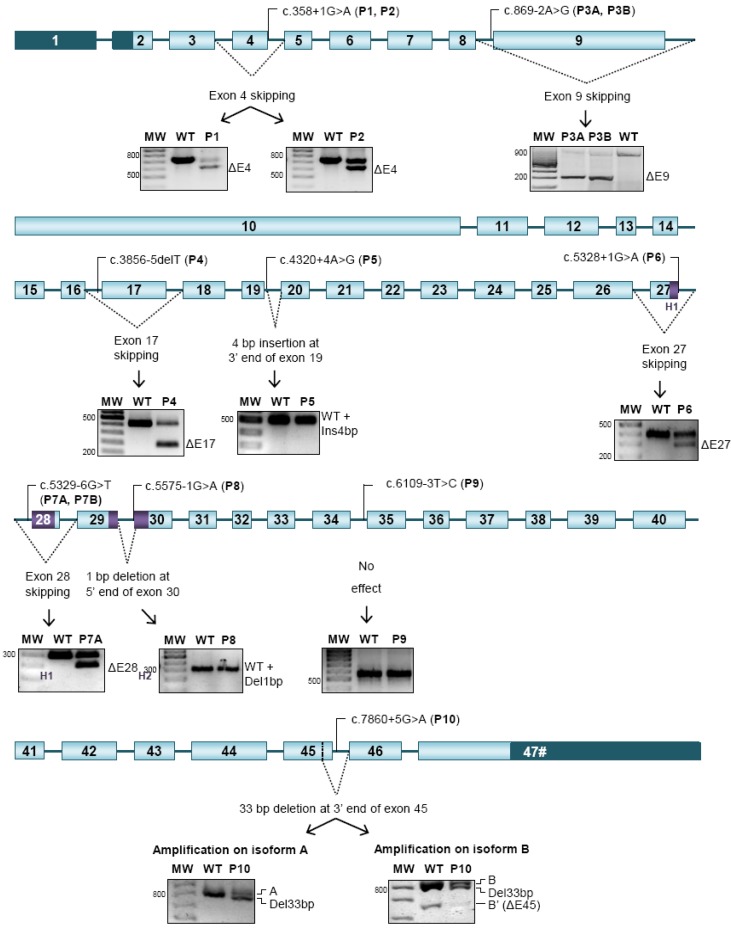
Overview about the localization of the mutations and its consequence upon transcript processing. Mutations are represented on isoform A. Boxes mean exons and dark lines mean introns. Dark boxes represent the untranslated region. The main functional domains in exons affected by splicing mutations are highlighted in violet (Undecapeptide repeat, H1 and H2 repeats in the HEAT (Huntington, Elongation Factor 3, PR65/A, TOR) domain). The localization of each mutation is shown above the gene, and their effect on splicing is shown under the gene. Dotted lines represent the aberrant splicing, and agarose gels with the analysis of the cDNA are shown. (MW: Molecular Weight, WT: Wild-Type, P1: Patient 1, P2: Patient 2, P3A: Patient 3A, P3B: Patient 3B, P4: Patient 4, P5: Patient 5, P6: Patient 6, P7A: Patient 7A, P7B: Patient 7B, P8: Patient 8, P9: Patient 9, P10: Patient 10).

By targeted Sanger sequencing of all 47 exons of the *NIPBL* gene we could identify nine allelic variants in our patients (Figure 1, Table 1) situated into the exon-intron junctions. Two of these variants have been previously reported: c.3856-5delT and c.6109-3T>C [14,17,18], while seven are new: c.358+1G>A, c.869-2A>G, c.4320+4A>G, c.5328+1G>A, c.5329-6T>G, c.5575-1G>A, and c.7860+5G>A. All of them were intronic, and four mapped into canonical AG/GT nucleotides affecting the conserved splice-donor or acceptor-site, respectively. The other five variants affected non-canonical nucleotides, from −6 to +5 positions.

The relevance of all nine splice-site mutations identified was investigated by specific RT-PCR using RNA isolated from fresh blood samples in order to identify aberrant splicing variants.

Patients 1 and 2 showed two transcripts (Figure 1), the normal band of 743 bp and another band of 615 bp corresponding to a deletion of exon 4. Patients 3A and 3B showed two transcripts, the normal band of 824 bp and another band of 197 bp consistent with a deletion of exon 9. Patient 4 showed two transcripts (Figure 1), the normal band of 449 bp and another band of 217 bp consistent with an exon 17-deletion. Patient 5 showed one PCR product (Figure 1), which contained both the normal transcript of 505 bp, and the aberrant transcript of 509 bp containing a 4 bp insertion at 3' end of exon 19. Patient 6 showed two transcripts (Figure 1), the normal band of 392 bp and another band of 289 bp representing an exon 27-deletion. Patient 7A showed two transcripts (Figure 1), the normal band of 304 bp and another band of 205 bp excluding exon 28. Patient 8 showed one PCR product (Figure 1), which contained both the normal transcript of 314 bp, and the aberrant transcript of 313 bp that shows a 1 bp deletion at exon 30 5' end. Patient 9 showed one PCR product of 578 bp (Figure 1) representing the normal transcript. Patient 10 was analyzed for isoforms A and B (Figure 1). In both, an aberrant transcript that contains a 33 bp deletion at 5' end of exon 45 in addition to the normal transcript could be detected. Moreover, also the transcript variant B’ was identified.

**Table 1:** Summary of molecular and clinical information in patients with splice site mutations.

Patient	Mutation	Intron	mRNA Change	*In Silico* Analysis	Predicted Protein Change	Clinical Data
**P1**	c.358+1G>A	4	Exon 4 skipping (128 bp)	Donor site disruption	p.(Ile77Metfs*5)	2-year-old boy with pre and postnatal growth retardation, developmental delay, typical facial features, heart valve stenosis and a feeding disorder.
**P2**	c.358+1G>A	4	Exon 4 skipping (128 bp)	Donor site disruption	p.(Ile77Metfs*5)	13-year-old boy with pre and postnatal growth retardation, intellectual disability with speech and motor delay, typical facial features, aortic stenosis and hirsutism.
**P3A**	c.869-2A>G	8	Exon 9 skipping (627 bp)	Acceptor site disruption	p.(Gly290_Lys498del)	3-year-old boy with pre and postnatal growth retardation, intellectual disability with speech and motor delay, typical facial features, autistic spectrum disorders, a feeding disorder, cryptorchidism and hirsutism.
**P3B**	c.869-2A>G	8	Exon 9 skipping (627 bp)	Acceptor site disruption	p.(Gly290_Lys498del)	36-year-old female with postnatal growth retardation, typical facial features, a feeding disorder, minor skeletal anomalies and hirsutism.
**P4**	c.3856-5delT [18]	16	Exon 17 skipping (232 bp)	Acceptor site disruption	p.(Asn1286Glufs*3)	23-year-old male with pre and postnatal growth retardation, typical features, intellectual disability, pyloric stenosis and a feeding disorder.
**P5**	c.4320+4A>G	19	Insertion of 4 pb at 3' end of exon 19	Donor site disruption and creation of a new cryptic donor site	p.(Phe1442Serfs*5)	7-year-old boy with postnatal growth retardation, intellectual disability with speech delay, typical facial features, deafness, GERD with a feeding disorder and small hands.
**P6**	c.5328+1G>A	27	Exon 27 skipping (103 bp)	Donor site disruption	p.(Met1743Serfs*16)	4-year-old boy with pre and postnatal growth retardation, developmental delay, typical facial features, pulmonar stenosis, cryptorchidism, bilateral aplasia of the ulna, dysplasia of distal humerus and monodactyly.
**P7A**	c.5329-6T>G	27	Exon 28 skipping (99 bp)	Acceptor site disruption	p.(Ile1777_Arg1809del)	9-year-old boy with has pre and postnatal growth retardation, intellectual disability with speech delay, typical facial features, hirsutism and small hands.
**P7B**	c.5329-6T>G	27	Exon 28 skipping (99 bp)	Acceptor site disruption	p.(Ile1777_Arg1809del)	40-year-old male with typical features, intellectual disability, hearing loss, ventricular septal defect and crytorchidism.
**P8**	c.5575-1G>A	29	Loss of 1 pb at 5' end of exon 30	Acceptor site disruption and creation of a new acceptor site	p.(Asp1859Ilefs*9)	1-year-old girl with pre and postnatal growth retardation, typical facial features, left ventricular hypertrophy, feeding disorder, horseshoe kidney, hirsutism, right amelia and small feet.
**P9**	c.6109-3T>C [16,17,18]	34	No effect	Maintenance of acceptor site strengh	No effect	26-year-old female with pre and postnatal growth retardation, intellectual disability with speech delay, typical facial features, deafness, feeding disorder, hirsutism, small hands and feet.
**P10**	c.7860+5G>A	45	Loss of 33 pb at 3' end of exon 45	Donor site disruption and activation of a cryptic donor site	p.(Val2610_Asp2620del)	8-year-old girl with speech delay, typical facial features and a feeding disorder.

Abbreviation: GERD, Gastroesophageal Reflux Disease; P1: Patient 1; P2: Patient 2; P3A: Patient 3A; P3B: Patient 3B; P4: Patient 4; P5: Patient 5; P6: Patient 6; P7A: Patient 7A; P7B: Patient 7B; P8: Patient 8; P9: Patient 9; P10: Patient 10.

Among the aberrant transcripts found, five cause a frameshift (patients 1, 2, 4, 5, 6, and 8), whereas three preserve the reading frame (Patients 3A, 3B, 7A, 7B, and 10) (Figure 1, Table 1).

The effect of all mutations was evaluated *in silico* with the programs Splice Site Prediction by Neural Network, HSF Matrices and MaxEnt [19,20].

For mutations located in the canonical position +1, which resulted in exon skipping, donor site disruption was confirmed (Table 1) with the three different programs used.

For c.5575-1G>A, which causes a loss of the first bp of exon 30, the three programs confirmed the disruption of the acceptor sequence, and predicted the generation of a new acceptor site one nucleotide downstream (Table 1).

For the rest of acceptor site mutations, in c.869-2A>G, c.3856-5delT, and c.5329-6T>G that resulted in exon skipping, acceptor site disruption was predicted (Table 2). However, the change c.6109-3T>C, which only showed the normal transcript, barely modified the acceptor site score strength (Table 2).

For donor site mutations, c.4320+4A>G leads to the insertion of four bps after exon 19. This mutation disrupted the original donor site in exon 19, but HSF Matrices and MaxEnt predicted the creation of a new one, four positions downstream (Table 1). In exon 45, the mutation c.7860+5G>A, which caused the deletion of 33 bp at the 3' end, disrupted the original donor site, but a cryptic donor sequence was found 33 nucleotides upstream (Table 1).

### 2.3. New Physiological Splicing Variants of NIPBL

**Figure 2:**
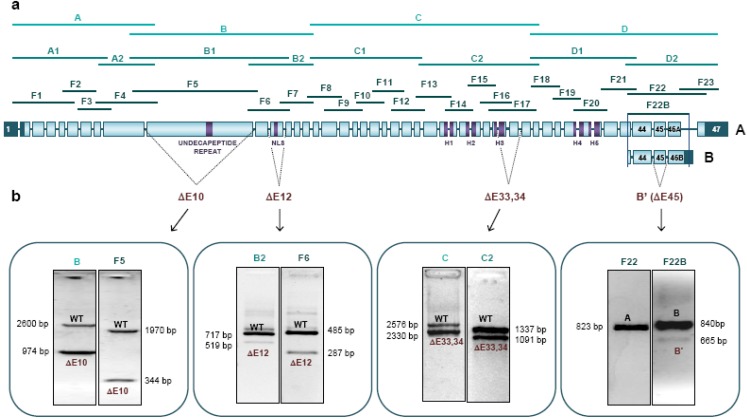
Systematic analysis of the physiological splicing of *NIPBL*. (**a**) Schematic representation of the two isoforms of the *NIPBL* gene and the three strategies used for the analysis of the physiological splicing. Boxes represent the exons and dark lines represent the introns. Exons 1–47 are shown for isoform A, and exons 44–46B are shown for isoform B. Dark boxes represent the untranslated region. The main functional domains are highlighted in violet (Undecapeptide repeat, Nuclear Localization Signal, H1–H5 domains in the HEAT repeats). Analysis of the physiological splicing has been performed amplifying the gene into four fragments (A–D, light green); into eight fragments (A1–D2, medium green); and into 23 fragments (F1–F23, dark green). F22B was used to specifically detect isoform B; (**b**) Physiological splicing variants of NIPBL found in human leukocytes. Four new variants with whole exon deletion have been found. Representative agarose gels are shown for each variant (WT: Wild-Type; ΔE10: exon 10 deletion; ΔE12: exon 12 deletion; ΔE33,34: exon 33 + 34 deletion; B’: exon 45 deletion on isoform B).

Using various combinations of different oligonucleotides specifically aligning to diverse exons in *NIPBL*, five splice variants could be amplified and confirmed on cDNA level (Figure 2b, Table 2). Interestingly, only one of which has been described as isoform B, while the remaining four have not been reported. All four new variants are the result of whole exon skipping, affecting exons 10, 12, 33 + 34, or 45. They have been submitted to GenBank, with the following accession numbers: KJ807789, KJ807790, KJ807791, and KJ807792.

**Table 2:** Splicing variants found in *NIPBL*.

Splicing Variant	mRNA Change	*In Silico* Analysis	Predicted Protein Change
ΔE10	Exon 10 skipping (1646 bp) c.1496_3121del	Exon 10 weak acceptor site	p.(Asp499_Lys1040del)
ΔE12	Exon 12 skipping (198 bp) c.3305_3502del	Exon 12 weak acceptor site	p.(Ser1102_Val1168delinsPhe)
ΔE33,34	Exons 33 and 34 skipping (246 bp) c.5863_6108del	Exon 33 weak acceptor site	p.(Leu1955_Ser2036del)
B	Exon 47 skipping (365 bp)and introduction of 211 bp of intron 46 c.8050_8415delins42	Exon 47 weak acceptor site	p.(Ser2864_Ser2904delinsValfs*13)
B’(ΔE45)	Exons 45 (175 bp) and 47 (365 bp) skipping and introduction of 211 bp of intron 46 c.[7686_7861del(;)8050_8415delins42]	Exons 45 and 47 weak acceptor site	p.(Lys2563Serfs*63)	

While the skipping of exons 10, 12, and 33 + 34 preserve the reading frame, loss of exon 45 cause a frameshift, as seen in variants B and B’ (Figure 2b, Table 2). A deletion of exon 10 (ΔE10) was found in amplifications B, B1, and F5. Deletion of exon 12 (ΔE12) was found in amplifications B1, B2, and F6 and skipping of the exons 33 + 34 (ΔE33 + 34) could be detected in amplifications C and C2. The deletion of exon 45 (ΔE45) was found in amplification F22B, specific for the known *NIPBL* isoform B, and defined as the new isoform B’. Specific amplification of the known *NIPBL* isoform A could not detect skipping of exon 45 (Figure 2b).

The strength of the exons involved in physiological splicing was evaluated using Splice Site Prediction by Neural Network. Exons 10, 12, 33, 45, and 47 showed very low acceptor site scores: (exon 10: 0.49, exon 12: 0.00, exon 33: 0.15, exon 45: 0.00, and exon 47: 0.41), and strong donor site scores (exon 10: 1.00, exon 12: 0.92, exon 33: 0.98, and exon 45: 0.96). Exon 34 was a well-defined exon, with acceptor and donor sites scores of 0.90 and 0.98, respectively (Table 2).

## 3. Discussion

In this work, we describe the first coordinated analysis of twelve CdLS–causing splice-site mutations in the *NIPBL* gene on DNA and RNA level as well as by *in silico* analyses. In order to properly assess aberrant splicing, we initially investigated the physiological *NIPBL-*splicing using RNA isolated from human leukocytes of normal controls.

Currently, two *NIPBL* splicing isoforms have been found in embryonic human tissues. Isoform A encodes a 2804 amino acid protein, while isoform B differs at in the 3'-part and codes for a 2697 aa NIPBL protein [12]. By our analyses we could identify and confirm the presence of four new isoforms (splice variants) in addition to isoform A and B in adult human leukocytes. One of these new isoforms, named Isoform B’, represents a transcript similar to isoform B excluding exon 45 which results in a shift of the reading frame. Systematic amplification of overlapping fragments could describe three new variants, carrying a deletion of exon 10 (ΔE10), exon 12 (ΔE12), and exons 33 + 34 (ΔE33,34), respectively (Figure 2b, Table 2). These findings were further supported by different *in silico* analyses indicating very weak splice acceptor-sites of exons 10, 12, 33, 45, and 47 (Table 2), with the exception of splice acceptor-site exon 34, which was predicted as strong splice-site [21]. We could show a combined skipping of exon 34 with the weak exon 33, which may drag the strong exon 34 during the splicing process, as previously reported for other genes [22,23].

Variants with deletion of exon 10, exon 12, and exons 33 + 34 maintain the reading frame, and could lead to functional proteins. The deletions of exons 10 and 12 affect the amino-terminal half of the gene, which is highly conserved in evolution [11]. However, the deletion of exons 33 + 34 affects the ancient carboxy-terminal half, which is conserved due to lower eukaryotes. Bioinformatic analyses suggest that variant ΔE10 could affect the undecapeptides repeat, which has been associated with transcriptional regulation; while variant ΔE12 would eliminate the predicted nuclear localization signal (NLS) of this protein [24]. On the other hand, variant ΔE33,34 would provoke the loss of the H3 domain in the HEAT repeats, which plays an important role in the interactions between NIPBL and the histone deacetylases (HDACs) 1 and 3 (Figure 2b) [25].

Among the twelve patients studied, we have detected nine splice-sites mutations, seven of them new, which represent 14% of the splicing mutations reported to date (Figure 1, Table 1). This kind of mutations show a random distribution across *NIPBL*, unlike nonsense and missense mutations, which tend to accumulate respectively in the first or in the last half of the gene [4,11].

Splicing mutations can result in different effects [26]. The most common is the skipping of the next exon. Here, this phenomenon has been found in mutations located in canonical nucleotides (c.358+1G>A, c.869-2A>G, and c.5328+1G>A), as well as in mutations affecting the polypyrimidine-rich tract (c.3856-5delT y c.5329-6T>G) (Table 1, Figure 1) [27].

Sometimes, splicing mutations can provoke the partial loss of the exon by activating cryptic splice sequences [28]. An example would be the mutation c.7860+5G>A, which disrupts the splicing donor sequence in exon 45. In this case, we would expect whole exon deletion, since exon 45 contains a weak acceptor sequence and skips physiologically yielding the variant B’ (Figure 2b). However, we have found an aberrant transcript with the deletion of 33 nucleotides at the 3' end of exon 45 (Figure 1). Bioinformatic analyses could predict a cryptic donor sequence at this position that is activated by the disruption of the original donor sequence (Table 1) [29]. Moreover, the mutation c.7860+5G>A could affect the ratio of physiological transcripts [23].

Eventually, splicing mutations can disrupt the original splice-sites and generate new ones [30,31], like in the mutations c.4320+4A>G (patient 5) and c.5575-1G>A (patient 8) (Figure 1, Table 1). In patient 5, an expansion of four nucleotides in exon 19 could be observed that disrupted the reading frame. This is in contrast to similar mutations previously reported (c.4320+2T>A and c.4320+5G>C), that result in an in frame skipping of exon 19 [4,32]. In patient 8, the mutation c.5575-1G>A was expected to cause the skipping of exon 30. Instead, it has generated an aberrant transcript corresponding to the deletion of the first nucleotide of exon 30. *In silico* tools predict that both mutations create new functional splice sites that were confirmed by sequencing of aberrant transcripts (Table 1).

Among the mutations analyzed here, c.6109-3T>C has not been considered as a functional splicing mutation since it has not generated aberrant transcripts (Figure 1), and was inherited from the healthy mother. Interestingly, this sequence variation has been previously reported in four CdLS patients [16,17,18] but was suggested to be not relevant for splicing by Leiden Open Variant Database (LOVD) [33].

Three of the splicing mutations analyzed here have produced transcripts with in frame deletions (c.869-2A>G (P3A and P3B), c.5329-6T>G (P7A and P7B), c.7860+5G>A (P10)), while five mutations have generated aberrant transcripts disrupting the reading frame (c.358+1G>A (P1 and P2), c.3856-5delT (P4), c.4320+4A>G (P5), c.5328+1G>A (P6) y c.5575-1G>A (P8)) (Figure 2). In *NIPBL*, frameshift mutations are often associated with more severe phenotypes as compared to mutations preserving the reading frame [4,15,16]. Splicing mutations identified in our patients seem to follow this tendency except patient 5 (c.4320+4A>G), who was classified as mild (Table 2). Thus, patients 1, 2, 4, 6, and 8 show severe pre- and postnatal growth delay, and P6 and P8 also show severe structural anomalies of the limbs (Table 1). Recently, in frame deletions and missense mutations affecting the HEAT domains have also been associated with severe phenotypes [15]. However, although patients 7A and 7B show an aberrant transcript with in frame deletion of a great part of the H1 domain, they had a mild phenotype with intellectual disability (Table 1).

## 4. Experimental Section

### 4.1. Patients and Controls

This study includes twelve patients diagnosed following the criteria from Kline *et al.* [1]. There are eight patients from Germany, and two familial cases, one from Poland (patients 3A and 3B, son and mother) and the other one from Spain (patients 7A and 7B, son and father). In accordance with the Declaration of Helsinki, the study had been approved by the ethics committee of the University of Lübeck, on November 2007 (reference number: 07-158). Patients’ parents have written individual informed consent to participate in the study. To perform the experiments, a pool of four control cDNAs from normal subjects was used.

### 4.2. DNA Extraction and Sequence Analysis

Genomic DNA was extracted from peripheral blood leukocytes using the standard procedures. The primers used to amplify the exons of the *NIPBL* gene and their splice junctions are provided on request. The PCR products obtained were purified with USB ExoSAP-IT PCR Product Cleanup (Affymetrix, Santa Clara, CA, USA) according to the manufacturer’s instructions, and sequenced on an ADN 3130 Genetic Analyzer (Applied Biosystems, Foster City, CA, USA).

The nucleotides of *NIPBL* cDNA were numbered according to the *NIPBL* isoform 1 (GenBank accession No. NM_000642). The mutation nomenclature was designated according to the Human Genome Variation Society [34] and confirmed by Mutalyzer [35]. In order to make data publicly available, mutations and associated phenotypic information were submitted to the Leiden Open Variant Database, Leiden, The Netherlands [14].

### 4.3. RNA Extraction and cDNA Synthesis

RNA was extracted from blood leukocytes using the PAXgene Blood RNA Kit (PreAnalytiX GmbH, Hombrechtikon, Switzerland) according the manufacturer’s instructions. Single-stranded cDNAs were synthesized with 500 ng of RNA from each patient using the First Strand Synthesis Kit (Thermo Fisher Scientific Inc, Waltham, MA, USA) with random hexamers, following the manufacturer’s protocol.

### 4.4. Identification of Splicing Variants

Physiological splicing variants were obtained using cDNA from a pool of four control individuals. Total *NIPBL* cDNA was amplified by PCR in overlapping fragments using three different approaches: dividing *NIPBL* into 4 fragments (A, B, C, and D), 8 fragments (A1, A2, B1, B2, C1, C2, D1, and D2), and 23 fragments (F1–F23). In this last strategy, an additional PCR (F22B) was performed to specifically amplify the isoform B (Figure 2a). Primers are provided on request.

To evaluate the aberrant splicing caused by mutations found in the *NIPBL* gene, specific PCRs that amplified the exons surrounding the mutations were performed on cDNA of each patient. Primers used are provided on request. The same reactions were carried out on cDNA from peripheral blood from a control individual.

For each PCR reaction, 2 μL of cDNA were used as a template in a total 20-μL mixture. Amplifications were carried out using 10 pmol of each PCR primer, 1× reaction buffer, 1.5 mM Mg_2_SO_4_, 200 μM dNTPs and 0.5 U Taq DNA polymerase. PCRs were performed in a thermocycler (Applied Biosystems) for 35 cycles at an annealing temperature of 56 °C.

PCR products obtained were analyzed by electrophoresis in 2% agarose gels, and all the bands were excised and purified with QIAEX Gel Extraction Kit (QIAGEN, Hilden, Germany), or purified with USB ExoSAP-IT PCR Product Cleanup (Affymetrix) when there was only a single band. The identity of each band was confirmed by sequencing on an ADN 3130 Genetic Analyzer (Applied Biosystems).

### 4.5. In Silico Splicing Analysis

First, we analyzed all *NIPBL* wild-type and mutated exons with Splice Site Prediction by Neural Network [19,36]. This bioinformatic tool assigns a strength score from 0.00 to 1.00 to acceptor (3' ss) and donor (5' ss) splice sites of each exon. It was used to evaluate the strength of affected exons, to predict disruption or creation of splice sites and to identify potential cryptic splice sites.

Later, we used a variety of tools integrated in the Human Splicing Finder [20] to perform a more exhaustive analysis of the exons affected by physiological/aberrant splicing. HSF [37] and MaxEnt [38] are tools that predict splice sites strength and can complement the data obtained from Splice Site Prediction by Neural Network.

## 5. Conclusions

In this study, he have performed the first systematic study of the physiological splicing of the *NIPBL* gene, that has allowed us to identify four new variants ΔE10, ΔE12, ΔE33,34, and B’, which should be kept in mind in order to assess the pathological splicing (Figure 2b). In addition, we have characterized eight splicing mutations, seven of which new, that means 14% of the reported mutations. The analysis of the RNA has ruled out that c.6109-3T>C is a splicing mutation, so its pathogenicity mechanism remains unclear (Figure 1). We also have confirmed that among the broad clinical variability that show the splicing mutations, the more severe phenotypes seem to associate to mutations generating frameshift transcripts.
